# Incorporating clinician insight and care plans into an audit and feedback initiative for antipsychotic prescribing to Medicaid-enrolled youth in Philadelphia

**DOI:** 10.1186/s12913-024-11029-5

**Published:** 2024-05-03

**Authors:** Molly Candon, Siyuan Shen, Aileen Rothbard, Abigail Reed, Mia Everett, Neal Demp, Melissa Weingartner, Oluwatoyin Fadeyibi

**Affiliations:** 1 Penn Center for Mental Health, Department of Psychiatry, Perelman School of Medicine, University of Pennsylvania, 3535 Market Street, 3rd Floor, Philadelphia, PA 19104 USA; 2 Leonard Davis Institute of Health Economics, University of Pennsylvania, Philadelphia, PA USA; 3 School of Social Policy and Practice, University of Pennsylvania, Philadelphia, PA USA; 4 Perelman School of Medicine, University of Pennsylvania, Philadelphia, PA USA; 5 Community Behavioral Health, Philadelphia, PA USA

**Keywords:** Antipsychotics, Medicaid, Off-label prescribing, Audit and feedback, Report cards

## Abstract

**Background:**

Audit and feedback (A/F), which include initiatives like report cards, have an inconsistent impact on clinicians’ prescribing behavior. This may be attributable to their focus on aggregate prescribing measures, a one-size-fits-all approach, and the fact that A/F initiatives rarely engage with the clinicians they target.

**Methods:**

In this study, we describe the development and delivery of a report card that summarized antipsychotic prescribing to publicly-insured youth in Philadelphia, which was introduced by a Medicaid managed care organization in 2020. In addition to measuring aggregate prescribing behavior, the report card included different elements of care plans, including whether youth were receiving polypharmacy, proper medication management, and the concurrent use of behavioral health outpatient services. The A/F initiative elicited feedback from clinicians, which we refer to as an "audit and feedback loop." We also evaluate the impact of the report card by comparing pre-post differences in prescribing measures for clinicians who received the report card with a group of clinicians who did not receive the report card.

**Results:**

Report cards indicated that many youth who were prescribed antipsychotics were not receiving proper medication management or using behavioral health outpatient services alongside the antipsychotic prescription, but that polypharmacy was rare. In their feedback, clinicians who received report cards cited several challenges related to antipsychotic prescribing, such as the logistical difficulties of entering lab orders and family members’ hesitancy to change care plans. The impact of the report card was mixed: there was a modest reduction in the share of youth receiving polypharmacy following the receipt of the report card, while other measures did not change. However, we documented a large reduction in the number of youth with one or more antipsychotic prescription fill among clinicians who received a report card.

**Conclusions:**

A/F initiatives are a common approach to improving the quality of care, and often target specific practices such as antipsychotic prescribing. Report cards are a low-cost and feasible intervention but there is room for quality improvement, such as adding measures that track medication management or eliciting feedback from clinicians who receive report cards. To ensure that the benefits of antipsychotic prescribing outweigh its risks, it is important to promote quality and safety of antipsychotic prescribing within a broader care plan.

**Supplementary Information:**

The online version contains supplementary material available at 10.1186/s12913-024-11029-5.

## Background

Antipsychotics play a key role in the treatment of irritability, impulsivity, and aggression in youth, but there are troubling side effects associated with their use, including weight gain and tardive dyskinesia [1–3]. These side effects can contribute to the onset of diabetes, cardiovascular disease, and high cholesterol levels, and should therefore be monitored carefully [4]. High rates of antipsychotic prescribing to some groups, such as foster care-enrolled youth, have raised additional concerns [5–7].

While the U.S. Food and Drug Administration (FDA) has approved antipsychotics to treat youth with conditions like autism and bipolar disorder [8], various studies have found that antipsychotics are frequently prescribed to youth for indications that are not approved by the FDA, such as attention deficit/hyperactivity disorder [9, 10].

As a result of these concerns, antipsychotic prescribing has been the target of numerous interventions in the U.S., including the introduction of prior authorization by insurers and the Substance Abuse and Mental Health Service Administration’s release of strategies to promote best clinical practices [11–13]. These efforts have coincided with a reduction in antipsychotic prescribing in recent years [14–16].

Another initiative that may have contributed to the decline in antipsychotic prescribing is audit and feedback (A/F), such as report cards, which can target low-value clinical practices [17]. A/F initiatives often include customized feedback, can be used with and without peer comparison, and have been shown to be effective at changing opioid and antibiotic prescribing behavior [18–20]. However, the impact of A/F initiatives is inconsistent, and systematic reviews suggest that A/F is most effective when baseline adherence to the wanted clinical practice is low [17].

Following opioids and antibiotics, A/F initiatives targeting the quality of antipsychotic prescribing behavior have been introduced in a variety of settings. Studies have found that report cards, a common type of A/F, can decrease antipsychotic prescribing rates for different patient populations, including adults with schizophrenia and youth with neurodevelopmental disorders [21–23]. Studies have also found that report cards can improve medication management associated with antipsychotic prescribing. For example, one study demonstrated a reduction in antipsychotic polypharmacy in community mental health centers and a second study found an increase in metabolic monitoring in a large outpatient clinic [24, 25]. Of note, a third study found no impact of report cards on high-dose antipsychotic prescribing or polypharmacy [26].

Given their inconsistent impact (and in the spirit of quality improvement), there is opportunity for operational enhancement in A/F initiatives like report cards. Yet few studies detail their implementation, such as the exclusion criteria of patients and clinicians, or the selection of prescribing measures. Beyond polypharmacy, report cards do not typically consider how antipsychotic prescribing is used within a broader care plan, such as the receipt of lipid and glucose testing or the concurrent use of behavioral health outpatient services. Another potential area of improvement is clinician engagement. Surveys of clinicians suggest that many view report cards as a “top-down” approach, and there is rarely opportunity for clinicians to provide their own feedback [27].

In this study, we describe the development and delivery of a report card that detailed antipsychotic prescribing to Medicaid-enrolled youth in Philadelphia. In addition to measuring aggregate antipsychotic prescribing behavior, the report card included different elements of the children and adolescents’ care plan, including polypharmacy, medication management, and the use of behavioral health outpatient services. We detail how the study sample and measures were selected, and also how this A/F initiative was embedded into a broader quality improvement effort in Philadelphia Medicaid. Finally, we evaluate the impact of the report card by comparing pre-post differences in prescribing measures for clinicians who received the report card with a group of clinicians who did not receive the report card.

## Methods

### Study setting and design

Philadelphia, Pennsylvania is the mid-Atlantic region of the U.S. and is considered one of the poorest large cities in the country [28]. Its population of over 1.6 million is 8% Asian, 44% Black, 15% Hispanic, and 45% White [28]. Medicaid is a government-funded insurer of millions of Americans, and its enrollees are predominately low-income adults, children and adolescents, and individuals with disabilities. Medicaid is the primary insurer for youth enrolled in foster care, sometimes called out-of-home care, which refers to children and adolescents under 18 years old who have been temporarily removed from their familial home and placed with either relatives or unrelated foster parents [28].

In Pennsylvania, Medicaid is operated at the county level and relies on managed care organizations. Community Behavioral Health (CBH), which is the sole behavioral health managed care organization in Philadelphia County, is responsible for paying for behavioral health outpatient and pharmacy services for approximately 700,000 Medicaid enrollees [29]. As a result, CBH has access to behavioral health, physical health, and pharmacy insurance claims that allow for a comprehensive set of prescribing measures to be included in a report card.

This study, which includes a cross-sectional analysis of prescribing measures used in the antipsychotic report card, was approved by the Institutional Review Boards at the University of Pennsylvania and the City of Philadelphia.

### Study sample

Our analysis began with all youth aged 0-17 years old who were enrolled in Philadelphia Medicaid and had at least 2 psychotropic prescription fills in 2019 (*n*=13,698), which were prescribed by 2,173 clinicians. Focusing on patients with 2 or more prescription fills aligns with other studies of medication adherence [30]. In 2019, there were 1,517 youth that had at least 1 antipsychotic prescription fill, which were prescribed by 553 clinicians. Of note, 85 youth received 1 antipsychotic prescription fill but no other psychotropic prescription fills and were therefore excluded from the analysis.

Nearly half of these clinicians were psychiatrists and neurologists (49%), with nurse practitioners and pediatricians making up another 30%. The distribution of specialties for the 553 clinicians is available in an appendix.

### Report card measures

Prescribing measures were based on information in the behavioral health, physical health, and pharmacy insurance claims for youth, which we are able to merge using a unique patient-level identifier.

The first measures we calculated were (1) the number of youth with at least one antipsychotic prescription fill and, of these, (2) the share of youth under age 14 with at least one antipsychotic prescription fill. An additional 6 measures were sourced from the Healthcare Effectiveness Data and Information Set (HEDIS) specifications as well as Pennsylvania’s quality monitoring initiative for foster-care enrolled youth [31]. They included: (3) the share of youth with an antipsychotic prescription receiving polypharmacy, defined as 3 or more psychotropic drug classes for 90 or more days; (4) the share of youth receiving at least 2 antipsychotics concurrently for 90 or more days; (5) the share of youth with an antipsychotic prescription who did not have an approved diagnosis in 2019 (we based this measure on the diagnoses listed in prior authorization forms for the Medicaid managed care organizations in Philadelphia, which included FDA-approved diagnoses like autism, bipolar disorder, and psychosis, as well as intellectual disability, Tourette’s syndrome, and conduct disorders); (6) the share of youth with an antipsychotic prescription who did not receive lipids and glucose testing in 2019; (7) the share of youth with an antipsychotic prescription who did not receive behavioral health outpatient services in 2019 [32]; and (8) the share of youth with an antipsychotic prescription who did not receive behavioral health outpatient services prior to their index antipsychotic prescription. Because behavioral health outpatient services were based on insurance claims, the service was available, offered, and not refused by patients and their families.

Rather than send report cards to all clinicians, we focused on those clinicians who prescribed antipsychotics to at least 10 Medicaid-enrolled youth (Table 1). Report card measures included peer comparison, which was based on the network average of the 553 clinicians who prescribed antipsychotics in 2019, including those who prescribed to fewer than 10 Medicaid-enrolled youth. Using the distribution of scores for each measure, clinicians were ranked normal, high, severe, or extreme compared to their peers on each measure, which was determined by the number of standard deviations above the network average. Since there were very few clinicians in the severe and extreme categories, the ranking was collapsed to normal versus high, which contained high, severe, and extreme categories. The high group therefore included clinicians who were 1 or more standard deviation above the mean.

**Table 1:** Study sample and exclusion criteria

#Clinicians with 1 or more antipsychotic prescription to Medicaid-enrolled youth	553
Exclude those with 0 high in measure 3,6, and 7 and 0 high in measure 4, 5, and 8	474
Exclude those with 0 high in measure 3, 6, 7 and 1 high in measure 4, 5, and 8	419
Exclude those with less than 10 youth with an antipsychotic prescription	80
Exclude missing and deactivated National Provider Identifier	78

In addition to excluding clinicians who prescribed antipsychotics to fewer than 10 Medicaid-enrolled youth from receiving a report card, we excluded clinicians who 1) had only normal measures; 2) had 0 high measures for measure 3, 6, and 7, and 1 high measure for measures 4, 5, and 8; and 3) clinicians who did not have a unique National Provider Identifier or had deactivated their licenses. The decisions regarding exclusion criteria were made by a quality team of child psychiatrists, pharmacists, and behavioral health liaisons at CBH.

After all exclusion criteria were met, report cards were mailed to 78 clinicians, who were representative of 35 unique agencies in Philadelphia and 1,580 clinician-patient relationships in which an antipsychotic prescription was filled (some patients had prescription fills from multiple clinicians). Among the 78 clinicians who received report cards, we categorized clinicians into one of three groups: (1) 22 clinicians who had a high categorization on 1 measure, for whom no written response was required; (2) 41 prescribers who had a high categorization on 2 or 3 measures, for whom a brief written response was required; and (3) 15 prescribers who had high categorizations on 4 or 5 measures, and were required to submit a root cause analysis (RCA). The RCA involved qualitative feedback and a clinical chart review of cases to provide rationale for their prescribing behavior. Clinicians only had to provide a response for measures where they performed above the network mean. Clinicians in the group requiring an RCA were given a 5-week initial timeframe to do so, with opportunities for extension and telephonic consultations if requested.

Finally, we evaluated the effectiveness of antipsychotic report cards by comparing antipsychotic prescribing patterns by clinicians before and after the report card was sent out (2019 vs. 2021). Of the 78 clinicians who received a report card, 64 continued prescribing antipsychotics to children in 2021, which allowed us to construct a pre-period and post-period; these prescribers serve as the intervention group. The comparison group is derived from the 475 remaining clinicians who did not receive report cards; of these, 143 continued prescribing antipsychotics to children in 2021. We estimated pre-post differences in prescribing patterns for clinicians receiving a report card, compared to those who did not. Differences were assessed using pairwise t-tests.

## Results

### Prescribing measures

**Fig. 1:**
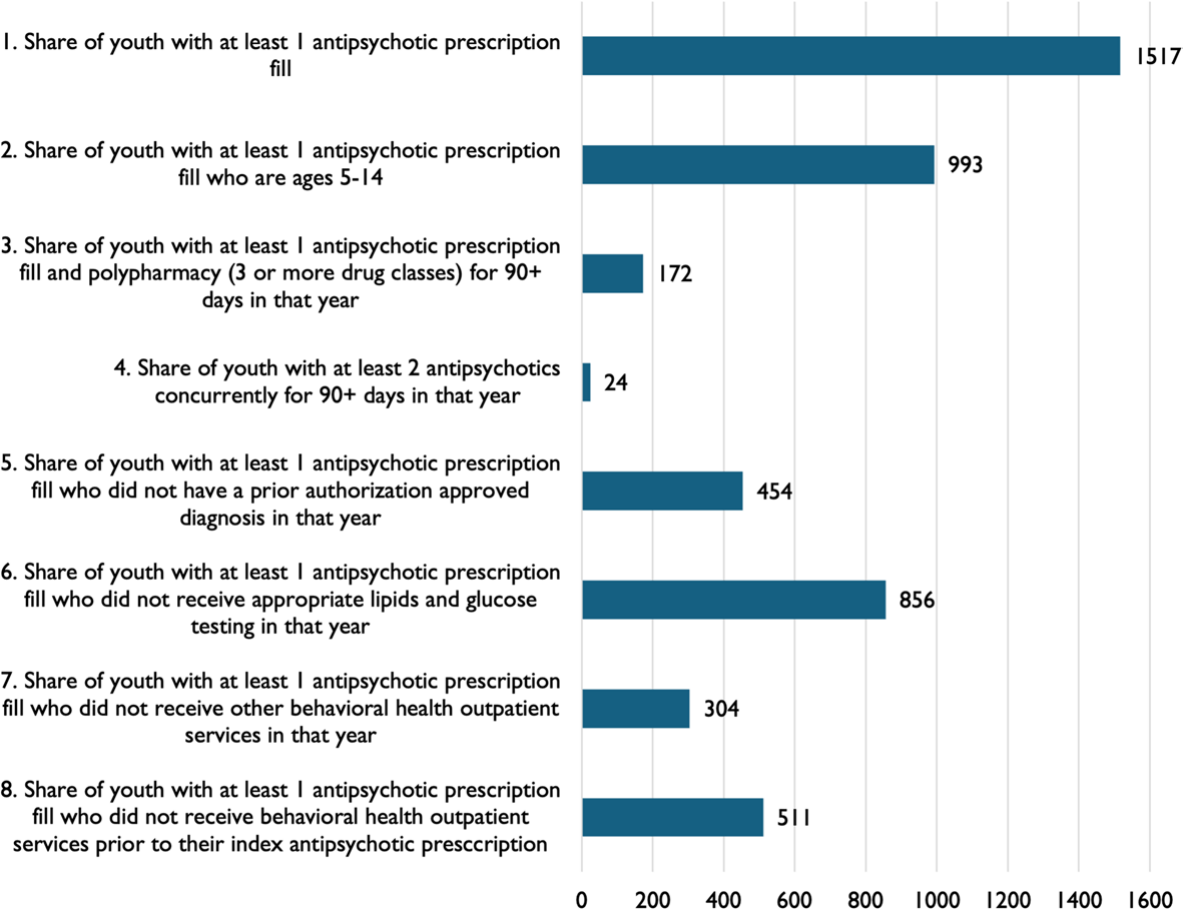
Number of Medicaid-Enrolled Youth for Each Prescribing Measure. Note. Antipsychotic prescriptions came from 553 unique providers

Of the 13,698 Medicaid-enrolled youth with at least 2 psychotropic prescription fills, 1,517 had at least 1 antipsychotic prescription fill (Fig. 1). Of these, 993 (65%) were aged 14 years or younger. Polypharmacy, as defined as youth who were receiving 3 or more psychotropic drug classes concurrently for 90+ days, including antipsychotics, occurred for 172 youth (11%), but only 24 were using 2 antipsychotics concurrently for 90+ days (2%). Antipsychotic prescribing for unapproved indications was more common, occurring among 454 youth (30%). In terms of the broader care plan, we found that 856 youth (56%) with an antipsychotic prescription in 2019 did not have lipids or glucose testing during the calendar year, 304 (20%) did not receive concurrent behavioral health outpatient services in 2019, while 511 (34%) did not have a behavioral health outpatient service prior to their index antipsychotic prescription.

### Feedback from clinicians

Report cards were constructed using network averages and the clinician-level measures, and were mailed in December 2020 (Table 2). Due to the tiered approach, no response was required of 28% of clinicians because they only had 1 high measure of the 6 measures included in the report card. Of the remaining 54 clinicians, 1 clinician was identified as never employed by the agency, 10 clinicians were identified as no longer employed by the agency, 6 clinicians were excused due to the inpatient population they served not being applicable, and 1 clinician did not provide a response. The quality team at CBH, which included child psychiatrists, pharmacists, and behavioral health liaisons, had to deem responses provided as satisfactory or not satisfactory. The determination was based on the completeness, clarity, and acceptability of the responses.

**Table 2:** Example of antipsychotic prescribing report card

**Measures**: **% of Medicaid-enrolled youth with**	**Network Mean**	**Dr. A %**	**Category**
Antipsychotic prescription receiving polypharmacy (3 or more drug classes) for 90+ days	11%	0%	NORMAL
At least 2 antipsychotics concurrently for 90+ days	2%	0%	NORMAL
Antipsychotic prescription who did not have a Pennsylvania prior authorization approved diagnosis in 2019	30%	42%	HIGH
Antipsychotic prescription who did not receive appropriate lipids and glucose testing in 2019	56%	92%	HIGH
Antipsychotic prescription who did not receive behavioral health outpatient services in 2019	20%	33%	HIGH
Antipsychotic prescription who did not receive behavioral health outpatient services prior to their index antipsychotic prescription	34%	42%	HIGH

Prior authorization diagnoses include autism, bipolar disorder, psychosis, conduct disorders, and intellectual disability

Approximately 40% of the 36 clinicians who needed to submit a written response provided concrete and time-based action plans to address the high measures that were brought to their attention. Nearly a dozen telephonic consultations occurred with clinicians who requested additional details, and the deadline for submission was extended into Spring 2021 to accommodate clinicians’ competing priorities given the persisting COVID-19 pandemic.

Report cards indicated that many youth who were prescribed antipsychotics were not receiving proper medication management or behavioral health outpatient services, and the written feedback from clinicians added important context to these findings. For example, several barriers to the medication management measure were cited, including the clinician’s software and office set-up not being favorable to proactively follow up after entering lab orders. Clinicians cited family members’ hesitancy, lack of understanding of the importance of medication management, and transportation barriers as reasons.

A common rationale for high polypharmacy measures was that clinicians frequently inherited patients with complex treatment regimens that they wouldn’t have initiated. They cited a hesitancy to taper antipsychotics, particularly when a patient’s presentation included trauma, multiple behavioral health diagnoses, and several failed medication trials. Clinicians also noted that parents and caregivers sometimes opposed the recommendation to taper, due to fear of their child regressing.

Some clinicians disagreed with our methodology and doubted the accuracy of the data used to determine their report card measures. To address this, members of CBH and the evaluation team held an open forum where they presented an overview of the report card, during which clinicians had the ability to learn about the initiative, ask questions, and provide comments. Clinicians requested that we utilize timelier data, provide more clarification about measures in the mailing, develop templates for the written responses, identify interventions that are directed to parents and caregivers, use affirming and supportive language to assuage concerns about penalties, and introduce an “office hours” for clinicians and agency leaders to ask questions about the data and methodology used in the report cards. Another insight was that that report cards tend to focus on inappropriate prescribing, and one clinician asked why report cards don’t account for appropriate prescribing as well. In other words, clinicians like to hear that they are doing well in relation to their peers, not just that they are doing poorly.

### Impact of the report card on prescribing measures

Overall, the number of youth receiving an antipsychotic prescription fell from 960 to 628 in the intervention group and increased from 496 to 506 in the comparison group between 2019 and 2021 (Table 3). We also found that the share of youth with 1 or more antipsychotic prescription fill who were between the ages of 5-14 fell significantly in both groups, whereas a significant drop in polypharmacy was only seen in the intervention group, where it fell from 14% to 10%. The share of youth with an unapproved diagnosis increased significantly in both groups, although the rise was greater in the comparison group (21% to 28% in intervention group compared to 28% to 43% in comparison group). There was a significant increase in the share of youth not receiving behavioral health outpatient services in the comparison group, from 23% to 36%.

**Table 3:** Difference in antipsychotic prescribing measure to Medicaid-enrolled youth, 2019 vs. 2021

	**Received a Report Card (*n*=64)**			**Did Not Receive a Report Card (*n*=143)**		
	**2019**	**2021**	***p*-value**	**2019**	**2021**	***p*-value**
1: Share of youth with at least 1 antipsychotic prescription fill	34%	34%	*p*=0.9812	29%	30%	*p*=0.7853
2: Share of youth with at least 1 antipsychotic prescription fill who are ages 5-14	70%	60%	*p*=0.0171	67%	57%	*p*=0.0302
3: Share of youth with at least 1 antipsychotic prescription fill and polypharmacy (3 or more drug classes) for 90+ days in that year	14%	10%	*p*=0.0160	14%	11%	*p*=0.2637
4: Share of youth with 2 antipsychotics concurrently for 90+ days in that year	2%	4%	*p*=0.3154	2%	4%	*p*=0.1517
5: Share of youth with at least 1 antipsychotic prescription fill who did not have a prior authorization approved diagnosis in that year	21%	28%	*p*=0.0320	28%	43%	*p*=0.0006
6: Share of youth with at least 1 antipsychotic prescription fill who did not receive appropriate lipids and glucose testing in that year	57%	49%	*p*=0.0554	52%	55%	*p*=0.4351
7: Share of youth with at least 1 antipsychotic prescription fill who did not receive other behavioral health outpatient services in that year	16%	20%	*p*=0.3590	23%	36%	*p*=0.0053
8: Share of youth with at least 1 antipsychotic prescription fill who did not receive behavioral health outpatient services prior to their index antipsychotic prescription	30%	33%	*p*=0.5726	37%	43%	*p*=0.7802
**Number of youth with at least 1 antipsychotic prescription fill**	960	628		496	506	

All providers prescribed at least one antipsychotic prescription in 2019 and in 2021

## Discussion

Audit and feedback (A/F) for clinicians can be effective at reducing antipsychotic prescribing. However, A/F initiatives like report cards rarely focus on other elements of a care plan, which may undermine their intended purpose to increase the overall quality of care delivered to patients. A/F initiatives tend to take a one-size-fits-all approach, despite the heterogeneity in clinical practice, and often have little opportunity for clinician engagement, which could allow for an "audit and feedback loop."

In 2020, Community Behavioral Health (CBH), which is the sole behavioral health managed care organization serving Medicaid enrollees in Philadelphia, Pennsylvania, introduced a report card focused on antipsychotic prescribing for youth. The report card, a common type of A/F, included a host of measures that captured other elements of the care plan, including polypharmacy (2+ antipsychotics taken concurrently and 3+ psychotropic medications taken concurrently), medication management (the receipt of lipids and glucose testing), and whether the youth engaged in behavioral health outpatient services. CBH opted for a tiered response, where written feedback was only requested from clinicians with multiple high measures.

The main objective of the report card was to encourage antipsychotic prescribing practices that are evidence-based, and the trends for several measures moved in the right direction. For example, there was a modest reduction in the share of youth receiving polypharmacy following the receipt of the report card, as well as a large reduction in the number of youth with 1 or more antipsychotic prescription fill. We did not find any change in the share of youth receiving behavioral health outpatient services along with their medication, which may require additional efforts.

The A/F initiative encompassed a data-informed approach for a cohort of thousands of patients and hundreds of clinicians, and CBH used the written and vocal feedback from clinicians to iterate the next iteration of the report card, which was mailed out in 2022. To address the clinicians’ concerns, we used timelier data and excluded the measure that required the outpatient behavioral health service to occur before the index prescription.

Of note, there were opportunities to leverage this A/F initiative to enhance other quality improvement initiatives. For example, CBH developed a “Provider Lab Tip Sheet” that was disseminated via email blasts, and which included a list of contracted laboratory providers serving Philadelphia Medicaid [33]. This allowed clinicians to more easily send their patients to a lab that accepted their insurance. It also pointed to best practices for labs and the diagnoses that are associated with quality monitoring.

We face several limitations, such as the size of the study sample, which was restricted to those clinicians who prescribed antipsychotics to 10 or more youth in 2019 and mostly comprised psychiatrists. Our findings may not generalize to report cards that target other provider types or clinical practices [34]. We are unable to capture whether patients adhered to their medications, a limitation when using pharmacy claims, nor whether outpatient services were clinically appropriate. Following other A/F initiatives, we use peer comparison as an incentive for behavior change, even when the benchmark may not be a reasonable one. E.g., ideally, the share of patients who receive lipids and glucose testing is higher than our estimated network mean.

There are additional questions and unintended consequences to consider, which are not examined in the present study. For example, a qualitative study of physicians in Sweden found that A/F initiatives were viewed as “top-down” interventions, which may undermine behavior change [23]. We were not able to demonstrate that our audit and feedback loop changed prescribing behavior more than a top-down intervention, at least empirically. Qualitative interviews with clinicians could unveil this, as well as how A/F initiatives interact with other quality interventions, such as prior authorization.

Despite these limitations, our study makes a number of contributions to the literature on A/F initiatives. We demonstrate the creation of an audit and feedback loop, whereby clinicians can engage in the process of developing a report card. This bidirectional, as opposed to top-down, intervention mean that clinicians can learn about their prescribing behavior from insurers and insurers can learn about mechanisms that influence prescribing behavior from clinicians. In Philadelphia, we will be using this approach for future interventions.

## Conclusion

This study described how an insurer or managed care organization can implement and iterate A/F initiatives. The report card for antipsychotic prescribing introduced in Philadelphia had a variety of measures, including polypharmacy, medication management, and behavioral health service utilization. It also employed a written feedback strategy and opportunities to involve clinicians in the quality improvement process. The impact of the report card was mixed: there was a modest reduction in the share of youth receiving polypharmacy following the receipt of the report card, while other measures did not change. However, we documented a large reduction in the number of youth with one or more antipsychotic prescription fill among clinicians who received a report card. The overarching goal of this report card, other A/F initiatives, and additional quality improvement efforts is to ensure that the benefits of antipsychotic prescribing outweigh its risks, and to promote quality and safety in antipsychotic prescribing within a broader care plan.

### Supplementary Information

**Supplementary Material 1.** 

## Data Availability

Our primary data source are Medicaid claims, which are not publicly accessible. The SAS code used to generate the findings is available upon request to Molly Candon, 3535 Market Street, 3rd Floor, Philadelphia, PA 19104, U.S., candon@upenn.edu.

## Abbreviations

A/F: Audit and feedback
FDA: Food and Drug Administration
CBH: Community Behavioral Health
HEDIS: Healthcare Effectiveness Data and Information Set
RCA: Root cause analysis
