# Supplementary material for: Incorporating clinician insight and care plans into an audit and feedback initiative for antipsychotic prescribing to Medicaid-enrolled youth in Philadelphia
Source: BMC Health Serv Res. 2024 May 3;24:574. doi: 10.1186/s12913-024-11029-5 (PMC11067128; doi:10.1186/s12913-024-11029-5)
Supplement: Supplementary file 1 — Supplementary Material 1. [file 12913_2024_11029_MOESM1_ESM.docx]

**Supplementary Table.** Specialty Distribution of Clinicians Used in Network Average for Antipsychotic Report Cards (N=553)

| *Specialty* | *Number of Prescribers* | *% of Prescribers* |
| --- | --- | --- |
| *Psychiatry & Neurology* | 270 | 48.82 |
| *Pediatrics* | 90 | 16.27 |
| *Nurse Practitioner* | 76 | 13.74 |
| *Student in an Organized Health Care Education/Training Program* | 29 | 5.24 |
| *Family Medicine* | 24 | 4.34 |
| *Internal Medicine* | 8 | 1.45 |
| *Physician Assistant* | 8 | 1.45 |
| *Emergency Medicine* | 7 | 1.27 |
| *Specialist* | 5 | 0.9 |
| *Hospitalist* | 4 | 0.72 |
| *Obstetrics & Gynecology* | 4 | 0.72 |
| *Registered Nurse* | 3 | 0.54 |
| *General Practice* | 2 | 0.36 |
| *Psychologist* | 2 | 0.36 |
| *Surgery* | 2 | 0.36 |
| *Clinic/Center* | 1 | 0.18 |
| *Clinical Nurse Specialist* | 1 | 0.18 |
| *Dentist* | 1 | 0.18 |
| *General Acute Care Hospital* | 1 | 0.18 |
| *Medical Genetics* | 1 | 0.18 |
| *Orthopedic Surgery* | 1 | 0.18 |
| *Otolaryngology* | 1 | 0.18 |
| *Pharmacist* | 1 | 0.18 |
